# Associations between maternal concern about child’s weight and related behaviours and maternal weight-related parenting practices: a cross-sectional study

**DOI:** 10.1186/s12966-018-0738-5

**Published:** 2018-10-24

**Authors:** Jess Haines, Katherine L. Downing, Lisa Tang, Karen J. Campbell, Kylie D. Hesketh

**Affiliations:** 1 Department of Family Relations and Applied Nutrition, University of Guelph, 50 Stone Rd E, Guelph, ON N1H 2W1 Canada; 2 Institute for Physical Activity and Nutrition (IPAN), School of Exercise and Nutrition Science, Deakin University, Geelong, Australia

**Keywords:** Maternal concern, Weight, Food parenting, Media parenting, Physical activity parenting

## Abstract

**Background:**

Parents influence their children’s weight-related behaviours through their parenting practices, which are often a focal point of obesity prevention interventions. This study examined associations of maternal concern about their child’s weight, dietary intake, physical activity, and media use with maternal food, physical activity, and media parenting practices.

**Methods:**

Mothers (*n* = 310) reported their level of concern regarding their child’s weight and related behaviours and their weight-related parenting practices when their child was 5 years of age as part of the Melbourne Infant Feeding Activity and Nutrition Trial (InFANT) Program. We used linear regression analyses with estimation by generalized estimating equations to examine associations of maternal concern and maternal parenting practices.

**Results:**

Slightly more than 60% of mothers reported at least one concern related to their children’s weight or related behaviours. Excessive media use was the most commonly endorsed concern among mothers (45.2%). Compared to mothers who were unconcerned about their child’s weight, mothers who were concerned about their child weighing too much reported higher levels of controlling feeding practices, i.e., restrictive feeding, lower levels of co-participation of physical activity, and higher levels of using media to control child behaviour. Mothers who were concerned their child weighed too little reported higher levels of controlling feeding practices, i.e., restrictive feeding, pressure to eat. Similarly, mothers who were concerned about their child’s eating (eating too much or too little) reported higher levels of controlling feeding practices. Mothers who were concerned about their child using too much media reported higher levels of using media to regulate their child’s behaviour and providing opportunities for their child to use media.

**Conclusion:**

Mothers who were concerned about their child’s weight, dietary intake, physical activity and media use reported higher levels of controlling parenting practices, i.e., pressure to eat, and lower levels of health promoting parenting practices, i.e., co-participation in physical activity. Longitudinal research is needed to elucidate temporal order and specific mechanisms of these associations.

## Background

Research shows that parents play a primary role in the development of their children’s diet, physical activity, and media (i.e., screen time) behaviours [1, 2]. Parenting practices are the strategies used by parents to influence their children’s behaviours [3, 4]. These strategies include modelling the behaviours, limiting or monitoring the behaviours, and providing encouragement or direct support for these behaviours. Given their strong influence on children’s behaviours, parenting practices are often a focal point of family-based obesity prevention interventions [3, 5].

Despite being identified as a key influence on children’s weight-related behaviours, limited research has explored determinants of these parenting practices. Understanding the factors associated with parenting practices is critical to inform interventions designed to change these practices. The majority of research in this area has focused on factors associated with maternal food parenting practices [6, 7], with few studies examining physical activity [8, 9] or media parenting practices [10]. One factor that has been shown to be associated with maternal food parenting is maternal concern about their child’s weight status. Studies among both pre-schoolers and school-aged children show that maternal concern about their child’s weight is positively associated with controlling, parent-led food parenting practices, such as pressure to eat and restriction [11–13]. These parent-led food parenting practices may have a negative effect on children’s weight as they are hypothesized to override rather than support children’s ability to regulate their intake [14]. It is unknown whether maternal concern about their child’s weight also results in more controlling practices related to other weight-related behaviours, such as physical activity and media parenting practices.

It is also unknown how maternal concern about their child’s individual physical activity or media behaviours are associated with their parenting practices related to these behaviours. Given the high level of societal stigma related to weight [15], it is possible that concern about child’s weight influences parenting practices differently than a parents’ concern about the child’s individual behaviours. Recent nationwide data suggest that Australian parents are concerned about their young child’s weight-related behaviours; in a recent national survey about child health, parents of preschool aged children identified excessive screen time and insufficient physical activity as two of their top five concerns about their child’s health [16].

This research aims to advance our understanding of factors that influence weight-related parenting practices by examining how concern about their child’s weight and related behaviours is associated with their food, physical activity and media parenting practices among Australian mothers of preschool children. Exploring this association may help inform interventions by identifying whether higher level of maternal concern about child weight-related behaviours could lead to improved food, physical activity or media parenting practices. Alternatively, our results may reveal that mothers who have high levels of concern may need additional support in implementing health promoting parenting practices.

## Methods

### Study design and sample

The Melbourne Infant Feeding Activity and Nutrition Trial (InFANT) Program was a 15-month cluster-randomized controlled trial to promote obesity-protective behaviours in early childhood that was implemented from 2008 to 2010 [17]. Using a two-stage random sampling design, 62 first-time parent groups were randomly selected from fourteen local government areas within a 60 km radius of the research centre (Deakin University in Burwood, Victoria, Australia). Groups with at least eight eligible families (English-speaking, first-time parents) who provided written informed consent, were randomized to either the intervention or control arm. A total of 528 mother-infant dyads were randomized.

The InFANT intervention involved six interactive sessions delivered during the regular meeting time of the first-time parents’ group. The intervention incorporated a range of modes of delivery (e.g. brief didactic sessions, take-home DVD and newsletters) and educational strategies focused on improving children’s dietary intake, limiting sedentary behaviour and provision of opportunities for physical activity, as well parental role-modelling of healthful behaviours. Participants in the control arm received quarterly newsletters on topics unrelated to the intervention, in addition to the usual care from their Maternal and Child Health nurse.

Mothers in both the intervention and control arms completed surveys at baseline and at child age approximately nine months, 20 months (end of intervention), and at follow-ups at three and a half years, and five years. This study uses the five-year survey data as the measures of interest were only assessed at that time. A total of 356 mothers completed the five-year survey. For this analysis, we excluded mothers who were missing all predictor and outcome variables of interest (*n* = 46). Thus, our final analytic sample included 310 mothers. Ethics approval was granted by the Deakin University Ethics Committee and the Victorian Office of Children.

### Measures

#### Outcomes variables

##### Maternal food parenting practices

Mothers reported on the following seven of the 12 subscales of the Comprehensive Feeding Practices Questionnaire (CFPQ): use of pressure in feeding, use of foods as rewards (i.e. offering favourite food/sweets for good behavior), restriction, modeling, monitoring, encouraging balance and variety, and use of food for emotion regulation [18]. These food parenting practices were selected as they were identified as most relevant for parents of preschool aged children and were shown to have adequate internal reliability (Cronbach’s > 0.60) in this sample [19]. Each item has a five-point response scale (score range 0–4) and subscale scores were calculated as recommended by taking the mean score of subscale items [18]. In addition to CFPQ subscales, mothers also reported covert control of child eating, which is defined as practices to control a child’s intake that cannot be detected by the child [20]. Covert control was scored using the same method as the factors from the CFPQ.

##### Maternal physical activity parenting practices

Purpose-designed items were used to assess maternal physical activity parenting practices. *Co-participation* was assessed with a single item that asked mothers how often in the past month did they engage in active play with their child. *Encouragement* was assessed with two items that asked mothers how often in the past month did they: a) Encourage their child to do something active; b).

Encourage their child to go outside to play. *Provide opportunities* was assessed with three items that asked mothers how often in the past month did they: a) Take their child for a ride on his/her bike or scooter; b) Take their child for a walk; c) Buy an item for their child to be active with (e.g. ball, bike/scooter). Each item had a six-point response scale (score range from 1 to 6). Subscale scores were calculated by summing the scores and taking the mean score of the subscale items.

*Restriction* was assessed with two items that asked mothers how much they agreed or disagreed with two statements: a) I often tell my child to sit still when he/she is being active outside; b) I often tell my child to sit still when he/she is being active inside. Each item had a four-point response scale (score range from 1 to 4). Restriction scores were calculated by summing the scores of these two items and taking the mean score.

##### Maternal media parenting practices

Purpose-designed items were used to assess maternal media parenting practices. *Co-participation* was assessed with a single item that asked mothers how often in the past month did they watch TV, videos or DVDs with their child. *Provide opportunities* was assessed with five items that asked mothers how often in the past month did they: a) Put the TV, a video or DVD on for their child to watch; b) Buy or rent a video or DVD for their child to watch; c) Switch the TV on when their child was in the room; d) Have the TV on when their child was playing; e) Have the TV on during dinner.

Each item had a six-point response scale (score range from 1 to 6). Subscale scores were calculated by summing the scores and taking the mean score of the subscale items.

*Using media for behaviour regulation* was assessed with two items that asked mothers how much they agreed or disagreed with two statements: a) I use TV to distract my child when he/she is being difficult; b) I use TV to keep my child occupied so that I can get things done. Each item had a four-point response scale (score range from 1 to 4). Scores for using media for behaviour regulation were calculated by summing the scores of these two items and taking the mean score of the items.

### Predictor variables

#### Maternal concern about child weight

Mothers were asked to indicate their level of concern about their child’s weight using a single item. Response options included: a) Very concerned about my child not weighing enough; b) A little concerned about my child not weighing enough; c) Not concerned about my child’s weight; d) A little concerned about my child weighing too much; and e) Very concerned about my child weighing too much. This item was coded into three groups: Not concerned about child’s weight (referent); Concerned about child not weighing enough (combine very concerned/ a little concerned); and Concerned about child weighing too much (combine very concerned/a little concerned).

#### Maternal concern about child dietary intake

Mothers were asked to indicate their level of concern about their child’s dietary intake using a single item. Response options included: a) Very concerned about my child not eating enough; b) A little concerned about my child not eating enough; c) Not concerned about my child’s eating; d) A little concerned about my child eating too much; and e) Very concerned about my child eating too much. This item was coded into three groups: Not concerned about child’s eating (referent); Concerned about child not eating enough (combine very concerned/ a little concerned); and Concerned about child eating too much (combine very concerned/a little concerned).

#### Maternal concern about child physical activity

Mothers were asked to indicate their level of concern about their child’s physical activity using a single item. Response options included: a) Very concerned about my child not being physically active enough; b) A little concerned about my child not being physically active enough; c) Not concerned about my child’s physical activity; d) A little concerned about my child being too physically active; and e) Very concerned about my child being too physically active. Because so few mothers (*n* = 6) indicated that they were concerned that their child was too physically active, we excluded those mothers and coded the other participants into two groups: Not concerned about child’s physical activity (referent); Concerned about child not being physically active enough (combine very concerned/ a little concerned).

#### Maternal concern about child media

Mothers were asked to indicate their level of concern about their child’s media use with two items; one asked about their child’s TV viewing and the other asked about their child’s use of computers and electronic games (including handheld games, tablets and smart phones). Response options were: a) Very concerned about my child not watching enough TV/ not using computers and electronic games enough; b) A little concerned about my child not watching enough TV/ not using computers and electronic games enough; c) Not concerned about my child’s TV viewing/ use of computers and electronic games; d) A little concerned about my child watching too much TV/ using computers and electronic games too much; e) Very concerned about my child watching too much TV/ using computers and electronic games too much. Because so few mothers (*n* = 3) indicated that they were concerned that their child did not watch enough TV/not use computers and electronic games enough, we excluded those mothers and coded the other participants into two groups: Not concerned about child’s TV viewing/use of computers and electronic games (referent); Concerned about child watching too much TV/ using computers and electronic games too much (combine very concerned/a little concerned).

### Statistical analysis

To adjust for covariates and to account for clustering by parent-group (level of recruitment), we used linear regression analyses with estimation by generalized estimating equations to assess cross-sectional associations between maternal concern regarding child weight and related behaviours, and maternal food, physical activity, and media parenting practices at child age five years. Because there were no differences in maternal concern or parenting practices between the intervention and control groups, we pooled data from both groups. We ran separate models for each predictor and outcome variable. We adjusted all estimates for intervention status, maternal education level, language spoken at home and maternal weight status.

## Results

The mean age of mothers was 37.5 (4.0) years (Table 1). English was the main language spoken at home and approximately 60% of mothers were university educated. Approximately 40% of mothers were overweight or obese. Just over 60% reported at least one concern related to their children’s weight or related behaviours, with the majority (37.1%) of those mothers reporting only one concern. Only 2% of mothers reported being concerned about their child’s weight and all three of their child’s weight-related behaviours. Media use was the most common concern among mothers with 45.2% of mothers reporting being concerned their child used media too much. Approximately 15% of mothers were concerned that their child was not active enough. More mothers were concerned that their child weighed too little (9.0%) than weighed too much (4.0%); similarly, more mothers were concerned that their child ate too little (18.4%) than ate too much (5.8%).

**Table 1:** Characteristics of mothers who participated in InFANT and completed the age-5 questionnaire, *N* = 310^a^

Mother Characteristic	N (%)
Age, mean (SD)	37.5 (4.0)
Maternal Education	
Low (≤ secondary school)	55 (17.7)
Medium (trade or certificate qualification)	66 (21.3)
High (university degree +)	189 (61.0)
Non-English speaker	9 (2.9)
Overweight or obese, BMI > 25 kg/m^2^	120 (40.8)
Concern about child’s weight	
Not concerned	268 (86.5)
Concern weighs too little	28 (9.0)
Concern weighs too much	14 (4.5)
Concern about child’s eating	
Not concerned	235 (75.8)
Concern eats too little	57 (18.4)
Concern eats too much	18 (5.8)
Concern about child’s physical activity	
Not concerned	260 (85.5)
Concerned not active enough	44 (14.5)
Concern about child’s media	
Not concerned	164 (54.9)
Concerned uses media too much	135 (45.2)
Number of concerns reported	
0	119 (38.4)
1	115 (37.1)
2	53 (17.1)
3	17 (5.5)
4	6 (1.9)
Child Characteristic	N (%)
Age, mean (SD)	5.0 (0.1)
Male children	168 (54.2)
Overweight/obese [34]	49 (15.7)

^a^numbers vary slightly due to missing data

In analyses adjusted for maternal education, language spoken at home, maternal weight status, and treatment condition, mothers who reported being concerned that their child weighed too little reported higher levels of pressuring their child to eat (β = 0.70, 95% CI: 0.30, 1.10; Table 2), restriction (β = 0.56; 95% CI: 0.20, 0.91), and use of food for rewards (β = 0.49, 95% CI: 0.08, 0.89) as compared to mothers who were not concerned about their child’s weight. In adjusted analyses, mothers who reported being concerned that their child weighed too much reported higher levels of restriction (β = 0.54, 95% CI: 0.20, 0.86) as compared to mothers who were not concerned about their child’s weight. Maternal concern about child’s weight, either too much or too little, was not significantly associated with any other food parenting practices.

**Table 2:** Associations between maternal concern about child weight and related behaviours and food parenting practices among mothers of 5-year old children, N = 310^a,b,c^

Maternal concern	Food parenting practices							
	Monitoring	Pressure	Restriction	Rewards	Modeling	Balance & variety	Emotional Regulation	Covert Restriction
	β (95% CI)	β (95% CI)	β (95% CI)	β (95% CI)	β (95% CI)	β (95% CI)	β (95% CI)	β (95% CI)
Child weight								
No concern	--- (ref)	--- (ref)	--- (ref)	--- (ref)	--- (ref)	--- (ref)	--- (ref)	--- (ref)
Weigh too little	−0.21 (− 0.64,0.23)	**0.70 (0.30, 1.10)**	**0.56 (0.20, 0.91)**	**0.49 (0.08, 0.89)**	0.07 (− 0.16, 0.31)	− 0.17 (− 0.37,0.02)	0.08 (− 0.14, 0.30)	−0.18 (− 0.48,0.12)
Weigh too much	− 0.18 (− 0.73,0.37)	−0.41 (− 0.91,0.01)	**0.54 (0.20, 0.86)**	−0.22 (− 0.77,0.31)	−0.09 (− 0.40,0.22)	−0.11 (− 0.29,0.07)	0**.**01 (− 0.35, 0.36)	0.31 (− 0.15,0.77)
Child eating								
No concern	--- (ref)	--- (ref)	--- (ref)	--- (ref)	--- (ref)	--- (ref)	--- (ref)	--- (ref)
Eat too little	**−0.44 (−0.82,-0.07)**	**0.44 (0.16, 0.71)**	**0.53 (0.22, 0.83)**	**0.46 (0.17, 0.75)**	0.02 (−0.17, 0.21)	−0.08 (− 0.20,0.04)	0.16 (− 0.02, 0.34)	−0.15 (− 0.41,0.12)
Eat too much	0.12 (− 0.31,0.56)	−1.10 (1.51,0.69)	**0.94 (0.60, 1.29)**	−0.28 (− 0.82,0.27)	0.06 (− 0.19,0.31)	−0.01(− 0.18, 0.16)	0.01(− 0.24, 0.26)	0.26 (− 0.08,0.59)

^a^All estimates are adjusted for maternal education, language spoken at home, maternal weight status, treatment condition

^b^When 95% CI does not include 0.0, the estimate and 95% CI are bolded

^c^Numbers vary slightly due to missing data

Mothers who reported being concerned that their child ate too little reported lower levels of monitoring their child’s intake (β = − 0.44, 95% CI: -0.82, − 0.07; Table 2) and higher levels of pressuring their child to eat (β = 0.44, 95% CI: 0.16, 0.71), restriction (β = 0.53, 95% CI: 0.22, 0.83), and using food for rewards (β = 0.46, 95% CI: 0.17, 0.75), as compared to mothers who were not concerned about their child’s eating. Mothers who reported being concerned that their child ate too much reported higher levels of restriction (β = 0.94, 95% CI: 0.60, 1.29) as compared to mothers who were not concerned about their child’s eating. Maternal concern about child’s eating, either too much or too little, was not significantly associated with any other food parenting practices.

Compared to mothers who reported that they were not concerned about their children’s weight, mothers who were concerned about their child weighing too much reported lower levels of co-participation of physical activity with their child (β = − 0.96, 95% CI: -1.63, − 0.29; Table 3). Maternal concern about child’s weight, either too much or too little, was not associated with any other physical activity parenting practices. Maternal concern about child not being active enough was not associated with any of the examined physical activity parenting practices (Table 3).

**Table 3:** Associations between maternal concern about child weight, child physical activity and physical activity parenting practices among mothers of 5-year old children, N = 310^a,b,c^

Maternal concern	Physical activity parenting practices			
	Co-participation	Encouragement	Opportunities	Restriction
	β (95% CI)	β (95% CI)	β (95% CI)	β (95% CI)
Child weight				
No concern	--- (ref)	--- (ref)	--- (ref)	--- (ref)
Weigh too little	0.01 (−0.50, 0.52)	**−**0.05 (− 0.48,0.37)	−0.09 (− 0.32, 0.14)	0**.**14 (− 0.11, 0.39)
Weigh too much	**−0.96 (−1.63, − 0.29)**	−0.28 (− 0.94, 0.38)	−0.11 (− 0.41, 0.20)	−0.02 (− 0.25, 0.21)
Child physical activity				
No concern	--- (ref)	--- (ref)	--- (ref)	--- (ref)
Not active enough	−0.49 (−1.00, 0.03)	−0.30 (− 0.78, 0.17)	−0.19 (− 0.38, 0.01)	0.07 (− 0.08, 0.22)

^a^All estimates are adjusted for maternal education, language spoken at home, maternal weight status, treatment condition

^b^When 95% CI does not include 0.0, the estimate and 95% CI are bolded

^c^Numbers vary slightly due to missing data

Compared to mothers who reported that they were not concerned about their children’s weight, mothers who were concerned about their child weighing too little reported higher levels of using media to regulate their child’s behaviour (β = 0.21, 95% CI: 0.04, 0.39; Table 4). Maternal concern about child’s weight, either too much or too little, was not associated with any other media parenting practices.

**Table 4:** Associations between maternal concern about child weight, child media use and media parenting practices among mothers of 5-year old children, N = 310^a,b,c^

Maternal concern	Media parenting practices		
	Co-participation	Behaviour Regulation	Opportunities
	β (95% CI)	β (95% CI)	β (95% CI)
Child weight			
No concern	--- (ref)	--- (ref)	--- (ref)
Weigh too little	−0.16 (− 0.56, 0.24)	**0.21 (0.04, 0.39)**	0.24 (− 0.07, 0.54)
Weigh too much	− 0.17(− 0.81, 0.47)	0.03 (− 0.38, 0.43)	−0.06 (− 0.39, 0.28)
Child media			
No concern	--- (ref)	--- (ref)	--- (ref)
Too much media	0.02 (−0.28, 0.32)	**0.40 (0.26, 0.52)**	**0.23 (0.05, 0.42)**

^a^All estimates are adjusted for maternal education, language spoken at home, maternal weight status, treatment condition

^b^When 95% CI does not include 0.0 (or 1.0 in the case of the OR), the estimate and 95% CI are bolded

^c^Numbers vary slightly due to missing data

Mothers who reported being concerned about their child using too much media reported higher levels of using media to regulate their child’s behaviour (β = 0.40, 95%CI: 0.26, 0.52; Table 4) and providing opportunities for their child to use media (β = 0.23, 95% CI: 0.05, 0.42). Maternal concern about child using too much media was not significantly associated with co-participation in screen time with their child.

## Discussion

Among this sample of 310 Australian mothers of preschool aged children, we found that level of maternal concern was relatively high with over 60% of mothers reporting at least one concern regarding their children’s weight and related behaviours. Overall, we found that maternal concern about a child’s weight or related behaviours was not associated with increased levels of health promoting parenting practices (e.g., encouragement of physical activity) and was, in fact, associated with lower health promoting parenting practices (e.g., lower rates of co-participation in physical activity) and higher rates of controlling parenting practices (e.g., restrictive feeding, using media to control behaviour).

Compared to those who were not concerned about their child’s weight or their children’s eating, we found that mothers who reported being concerned about their children’s weight or eating reported higher levels of controlling, parent-led feeding practices. These results support previous research that found mothers’ concern about children’s weight resulted in more frequent use of pressure to eat [11, 21] and restrictive feeding practices [11, 12, 22]. In their longitudinal study with over 4800 children, Jansen and colleagues also found that when mothers perceived that their child ate very little they pressured their child to eat more [23]. Taken together, these findings indicate that maternal concern about their child’s weight status and dietary intake may lead to parents using controlling feeding practices, which have been shown to have a negative effect on a child’s relationship with food [24] and result in poor self-regulation of eating, which can lead to excessive weight gain [14].

We found that mothers who were concerned about their child weighing too much reported lower levels of co-participation in physical activity. However, there were no differences in health promoting physical activity parenting practices, such as encouraging or providing opportunities for child’s physical activity. This suggests that concern about child’s weight does not appear to translate into mothers engaging in health promoting parenting practices regarding child physical activity. While some researchers have argued that identification of their child’s weight as a problem is a prerequisite to parental behaviour change [25–28], our findings as well as those from previous research [12, 29], suggest that parental concern about their child’s weight does not lead to health promoting weight-related parenting practices. Among a sample of 314 American parents (92% mothers), those who recognized that their child was overweight or obese were no more likely to engage in health promoting parenting practices, such as increasing access to healthful foods or encouraging their child to be physically active, than parents who did not recognize their child was overweight or obese [29]. In their study with 84 parents (55 mothers) of six-year olds, Norman and colleagues found that whilst parents reported wanting to play and be physically active with their children, everyday stress and time constraints made it difficult to be active with their children on a regular basis [30]. These results suggest that to effectively increase parent engagement in health promoting parenting practices, health practitioners and health interventions should provide families with practical strategies on how to implement such practices within the context of their families’ busy lives rather than heighten parental concern about child’s weight or related behaviours.

Similar to our findings regarding mother’s concern about their child’s weight, our results suggest that maternal concern about screen time does not translate into higher levels of health promoting parenting practices; in fact, mothers who were concerned about their child’s media use were more likely to provide opportunities for their child to use media and reported higher levels of using media to regulate their child’s behaviour. It is possible that mothers’ report of concern regarding their children’s media use is a reflection of mothers’ low confidence, i.e., self-efficacy, in their perceived ability to change their child’s behaviour, i.e., mother’s low confidence to impact children’s media use leads to higher levels of concern regarding the behaviour. This low confidence may be the result of low knowledge or skills to implement health promoting parenting practices. The ubiquitous nature of screen use in the current culture, among both children and their parents, may also influence mothers’ confidence and their implementation of health promoting parenting practices regarding their children’s screen time. Although the cross-sectional nature of this study precludes us from determining the temporal order of the association between maternal concern and their parenting practices, our results suggest that high maternal concern regarding children’s weight-related behaviours may be helpful in identifying families who require additional supports to adopt health promoting weight-related parenting practices.

In addition to the cross-sectional nature of this study, additional limitations that should be considered when interpreting our study results include the use of brief, maternal self-report measures of their parenting practices. This may result in social-desirability bias or possible misclassification, which would likely bias estimates towards the null. Second, this study includes only mothers. Given the emerging evidence of the key role father’s play in the development of their children’s weight-related behaviours [31–33], future studies should aim to understand father’s concern regarding their children’s weight and related behaviours and how this concern is associated with father’s weight-related parenting practices. Lastly, a high proportion of mothers in this study were university educated, which may reduce the generalizability of our findings. It is possible level of concern about children’s weight and related behaviours is higher among more parents with higher education or income; however, the national survey of parental concern about their child’s health found that excessive screen time and inadequate physical activity were identified by both lower (<$1000/week) and higher income (>$1000/week) parents as a top five health concern [16], suggesting that level of concern regarding these behaviours may be similar across socio-economic groups.

## Conclusion

Our cross-sectional analysis found that maternal concern about a child’s weight or related behaviours was not associated with increased levels of health promoting weight-related parenting practices, but rather weight-related parenting practices that are counterproductive to the goal of healthy behaviours among children. These findings suggest that simply informing mothers that their child’s weight, dietary intake, physical activity, or screen time behaviours are putting their children at risk for poor health outcomes may not lead to more health promoting parenting practices. Instead, a shift in the way health care professionals and government supported health programs assist mothers in implementing health promoting parenting practices, through effective tools and concrete strategies that guide change, may be beneficial. As this was the first study to examine how concern about children’s media and physical activity behaviours are associated with related parenting practices, further longitudinal research is needed to elucidate the temporal order and potential mechanisms by which parental concern influences parenting practices.

## Abbreviations

BMI: Body Mass Index
CFPQ: Comprehensive Feeding Practices Questionnaire
DVD: Digital Versatile Disk
InFANT: The Melbourne Infant Feeding Activity and Nutrition Trial
TV: Television
